# Addressing potentially inappropriate prescribing in older patients: development and pilot study of an intervention in primary care (the OPTI-SCRIPT study)

**DOI:** 10.1186/1472-6963-13-307

**Published:** 2013-08-14

**Authors:** Barbara Clyne, Marie C Bradley, Carmel M Hughes, Daniel Clear, Ronan McDonnell, David Williams, Tom Fahey, Susan M Smith

**Affiliations:** 1 HRB Centre for Primary Care Research, Royal College of Surgeons in Ireland (RCSI), Beaux Lane House, Lower Mercer Street, Dublin, Ireland; 2 School of Pharmacy, Queen’s University Belfast (QUB), University Road, BT7 1NN, Belfast, Northern Ireland; 3 Department of Geriatric and Stroke Medicine, Royal College of Surgeons in Ireland (RCSI), Beaumont Hospital, Beaumont Road, Dublin, Ireland

**Keywords:** Medical research council framework, Multifaceted intervention, Potentially inappropriate prescribing, Randomised controlled trial

## Abstract

**Background:**

Potentially inappropriate prescribing (PIP) in older people is common in primary care and can result in increased morbidity, adverse drug events, hospitalizations and mortality. The prevalence of PIP in Ireland is estimated at 36% with an associated expenditure of over €45 million in 2007. The aim of this paper is to describe the application of the Medical Research Council (MRC) framework to the development of an intervention to decrease PIP in Irish primary care.

**Methods:**

The MRC framework for the design and evaluation of complex interventions guided the development of the study intervention. In the development stage, literature was reviewed and combined with information obtained from experts in the field using a consensus based methodology and patient cases to define the main components of the intervention. In the pilot stage, five GPs tested the proposed intervention. Qualitative interviews were conducted with the GPs to inform the development and implementation of the intervention for the main randomised controlled trial.

**Results:**

The literature review identified PIP criteria for inclusion in the study and two initial intervention components - academic detailing and medicines review supported by therapeutic treatment algorithms. Through patient case studies and a focus group with a group of 8 GPs, these components were refined and a third component of the intervention identified - patient information leaflets. The intervention was tested in a pilot study. In total, eight medicine reviews were conducted across five GP practices. These reviews addressed ten instances of PIP, nine of which were addressed in the form of either a dose reduction or a discontinuation of a targeted medication. Qualitative interviews highlighted that GPs were receptive to the intervention but patient preference and time needed both to prepare for and conduct the medicines review, emerged as potential barriers. Findings from the pilot study allowed further refinement to produce the finalised intervention of academic detailing with a pharmacist, medicines review with web-based therapeutic treatment algorithms and tailored patient information leaflets.

**Conclusions:**

The MRC framework was used in the development of the OPTI-SCRIPT intervention to decrease the level of PIP in primary care in Ireland. Its application ensured that the intervention was developed using the best available evidence, was acceptable to GPs and feasible to deliver in the clinical setting. The effectiveness of this intervention is currently being tested in a pragmatic cluster randomised controlled trial.

**Trial registration:**

Current controlled trials ISRCTN41694007

## Background

Potentially inappropriate prescribing (PIP) is a term used to describe a number of suboptimal prescribing practices, particularly the use of medicines that introduce a greater risk of adverse drug-related events where a safer, as effective alternative is available to treat the same condition [1]. PIP is common in older people and can result in increased morbidity, adverse drug events, hospitalizations and mortality [2-4]. PIP is usually measured using either explicit (criterion-based) or implicit (judgment-based) tools. Using a recently developed explicit process measure, the Screening Tool of Older People’s Prescriptions (STOPP) criteria, the prevalence of PIP in older people (aged ≥ 70 years) in Ireland has been estimated at 36%, associated with an expenditure of over €45 million in 2007 (or 9% of expenditure on pharmaceuticals in that age group) [5]. Studies have reported a prevalence of 35% to 47% in hospitalized older patients [6,7] and 73% in nursing homes [8]. The clinical and economic burden of PIP is therefore an important public health concern and it is important to minimize PIP where possible to increase patient safety and encourage cost-effective prescribing behaviour.

Changing prescribing behaviour such as PIP, is a complex and challenging task. Several strategies have been used to alter prescribing practices with variable results, with no one interventional strategy proving to be consistently effective [2,9-11]. Multifaceted interventions combining a number of techniques within an intervention [10], may be more effective than any one single intervention in altering prescribing as multiple elements can target different aspects of behaviour [2,12,13]. Multifaceted interventions are complex interventions, involving multiple targets (e.g. patients, clinicians) and various active components that may act both independently and interdependently [14,15]. In targeting PIP, the impact of multifaceted interventions to date has been mixed [9,10,16]. However, such approaches are still widely used to address prescribing in research. As complex interventions can be difficult to define and develop, the United Kingdom Medical Research Council (MRC) published a framework to guide researchers in this task [15]. Initially, this framework followed a five phase, linear approach. This has subsequently been updated to be more flexible, reflecting that research on the context, the intervention and the evaluation may be conducted simultaneously rather than sequentially [14,17]. In the revised framework, the development stage aims to gain an understanding of the problem, the intervention and the evaluation. Both feasibility testing and piloting are used to identify key uncertainties of the study design prior to the full randomised control trial (RCT) such as acceptability, compliance, feasibility and delivery of the intervention and recruitment and retention. Once the underlying problem has been examined and a credible intervention developed, the definitive trial may be undertaken to determine the effectiveness of the intervention and plans for future implementation put in place.

## Methods

**Figure 1:**
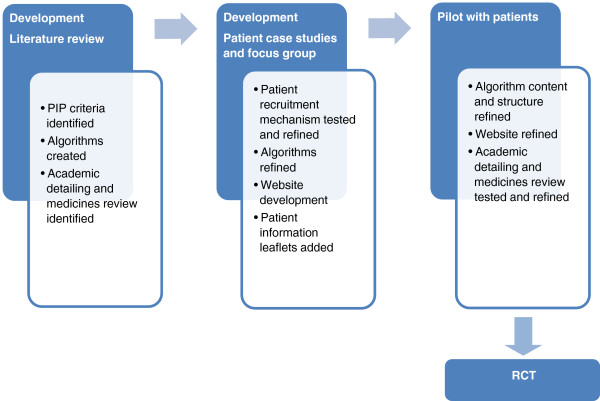
**Flowchart of intervention development adapted from MRC framework.** Abbreviations – PIP (Potentially Inappropriate Prescribing); RCT (Randomised Controlled Trial).

The aim of this paper is to describe the application of the early stages of the MRC framework to the development of a multifaceted intervention aimed at reducing PIP in Irish primary care. The specific methods and results of each of the stages of the MRC framework are presented separately, in sequence. In the development stage, literature was reviewed and combined with information obtained from experts in the field using a consensus based methodology and patient cases to define the main components of the intervention. In the pilot stage, five GPs tested the proposed intervention. Qualitative interviews were conducted with the GPs to inform the development and implementation of the intervention for the main randomised controlled trial. NVivo was used to assist with organizing the data for analysis. The aims and methods used are summarised in Table 1. The development and pilot process is summarised in Figure 1.The wider effectiveness and acceptability of the intervention is currently being tested in the OPTI-SCRIPT study (OPTImizing PreSCRIbing for Older People in Primary Care, a clusTer randomized controlled trial) which is described in detail elsewhere [18].

**Table 1:** Summary of aims and methods

**Process**	**Aims**	**Methods**
Identifying the evidence base and theory	To explore the empirical and theoretical evidence relating to PIP and interventions and identify intervention components	Literature search of selected databases and literature review
Modelling	To identify PIP criteria to include in the study	Consensus based methodology
	To identify alternative treatment options	Consensus based methodology
	Testing components of the intervention with GPs	Patient case studies
	Testing patient identification mechanism	Patient case studies
	Assessing GP perspectives on intervention	Focus group
Pilot	To test the intervention	GPs conducting medicines review
	To evaluate GP perspectives on intervention	Semi-structured interviews with GPs

## Results

### Development phase

The development phase was aimed at understanding the problem, the evidence to support intervention development and the evaluation, through reviewing the existing literature, identifying theory and modelling processes and outcomes.

### Evidence base and theory

A literature search was conducted to identify PIP criteria and the empirical and theoretical evidence relating to intervention research and altering prescribing practice (initially conducted in October 2011 and updated in February 2012). Pubmed, EMBASE, the Cochrane Library of Systematic Reviews and Web of Knowledge were searched and ‘grey literature’ was searched using SCIRUS and Lenus. Combinations of MeSH terms and keywords were used, including inappropriate prescribing; appropriate prescribing; older people; aged; elderly; controlled clinical trials as topic; intervention studies; prescribing interventions; primary care. Additional publications were identified by a manual search of the reference lists of relevant studies and review articles. No time period was applied but the search was limited to English language and human studies.

The PIP literature was used to select PIP criteria, as described in the modelling section below. In terms of interventions to alter prescribing practices, several strategies have been used with variable results. These include academic detailing, computerised decision support systems (CDSS), audit and feedback, the use of multidisciplinary teams, and interventions by pharmacists conducting a medicines review [10,19-23]. These individual interventions have shown mixed results in improving the quality of prescribing in older patients and where they are effective, the effect sizes tend to be small to modest. Multifaceted interventions, an approach which combines a number of techniques within a single intervention [10], may be more likely to work than any one single intervention in altering prescribing as multiple elements can target different aspects of behaviour [2,12,13]. In targeting PIP, the impact of multifaceted interventions to date has been mixed [9,10,16]. However, such approaches are still widely used to address prescribing in research.

The use of behavioural theory has also been highlighted as an important factor in designing interventions [15]. Despite a large body of literature on behaviour change theory, no single theoretical model has been universally accepted and the theoretical underpinnings of interventions are not consistently described and operationalised in the literature [14,24]. Some question the usefulness of such theory in intervention design. Bhattacharyya et al. question the lack of clarity as to how to translate theory reliably into intervention design and why any one theory should be given primacy over another [25], while Oxman et al. argue that less rather than more focus is needed on theory [26]. Work is on-going to develop a universally agreed method of specifying intervention components aimed at changing behaviour [27]. In the absence of clear guidance in the application of theory in intervention research, it is useful to have a framework for organising the factors that may influence changes in clinical behaviours. One such model identified in the literature was the PRECEDE-PROCEDE model. This is a planning model which organises the influencing factors into three categories – predisposing factors, enabling factors and reinforcing factors [28]. The PRECEDE model was used to organise the evidence from the literature - predisposing factors included GP knowledge of PIP, awareness of PIP and PIP criteria, and belief in value of PIP criteria. Enabling factors included GP learning, resources available to GP, GP time, patient expectation and reinforcing factors related to available incentives such as feedback of research findings and participation in continuing professional development programmes.

From the evidence base, two initial intervention components were identified. The first was academic detailing to address GP knowledge of PIP and PIP criteria, which was identified in the literature as an important barrier to appropriate prescribing [29]. The second was conducting a medicines review which has been identified as a strategy to address PIP [10,30,31]. Pharmacists were identified as the best candidate to facilitate the academic detailing with GPs to provide both education on PIP and advice and support to enable GPs conduct medicines reviews [23,32,33]. The form and content of these components was conceptualised and expanded through the modelling process.

### Modelling

The MRC guidelines recommend the use of both quantitative and qualitative methodologies in the design and evaluation of an intervention. Qualitative methods can contribute in several ways to the design and refinement of an intervention by identifying intervention components in need of further refinement, barriers or facilitators to implanting an intervention and involving users in the development process [34,35]. In this research, qualitative research methods contributed to the refinement of the intervention through the use of a focus group during modelling and semi-structured interviews in the pilot stage.

During the modelling stage, the findings from the literature were combined with information obtained from experts in the area, including general practitioners (GPs), pharmacists and a specialist in clinical pharmacology and medicine for the elderly, firstly using a consensus-based methodology and secondly, using patient case studies followed by a focus group, both of which are described below. These processes allowed us to identify PIP for inclusion in the study and alternative therapy options. It also allowed us to test the acceptability of components of the intervention with GPs and provided the opportunity to test methods of patient identification and recruitment.

### Consensus-based methodology – selecting PIP criteria and alternative treatment options

A preliminary list of individual PIP criteria for inclusion in the study was compiled from the most commonly cited existing published criteria, as identified from the literature search. These included the Beers criteria [36], the STOPP criteria [37], The McLeod criteria [38], the Improving Prescribing in the Elderly Tool (IPET), [39] the Assessing Care of the Vulnerable Elder (ACOVE) [40], and the Prescription Peer Academic Detailing (Rx-PAD) study (see Table 2 for summary of these criteria) [41]. Duplicate criteria were removed from the list and the prevalence of each individual PIP criteria in Ireland was sourced from the literature where available. The initial list consisted of 122 individual criteria. A total of 42 criteria were removed as not being applicable to the Irish setting (i.e. where specific medications were unavailable and not being considered to be an absolute contra indication as per the British National Formulary (BNF) or Irish Medicines Formulary (IMF)) resulting in a list of 80.

**Table 2:** Summary of selected PIP criteria

**Criteria**	**Year**	**Country of origin**	**Number of criteria**	**Target group**	**Method of development**
McLeod	1997	Canada	38	General population ≥ 65	Delphi consensus method
IPET	2000	Canada	14	General population ≥ 70	Based on McLeod, validated in a geriatric unit
Beers	2003*	USA	68	General population ≥ 65	Delphi consensus method
Rx-PAD	2006	Norway	14	General population ≥ 70 years	Based on literature and Delphi consensus method
ACOVE	2007	USA	392	Community- dwelling ≥ 65 at greater risk of death/functional decline	Delphi consensus method
STOPP	2010	Ireland	65	General population ≥ 65	Delphi consensus method

* Note: The Beers criteria were first developed in 1991 and updated in 1997, 2003 and most recently in 2012. The 2003 version was included in this study.

A panel consisting of two GPs (TF, SS), two pharmacists (CH, MB) and a physician (DC) was convened by the research manager (BC). In the first round, the list of 80 PIP criteria was circulated to the panel for independent assessment as to which criteria were suitable for inclusion in this study. The inclusion criteria were:

• Prevalence of indicator

• Clinical significance of indicator

• Evidence base to support inclusion.

**Figure 2:**
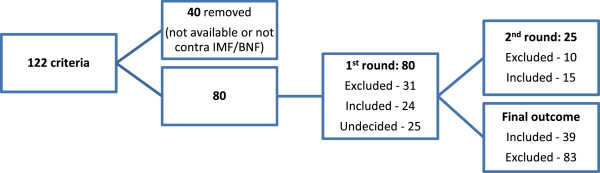
**PIP criteria review process.** Abbreviations – BNF (British National Formulary); IMF (Irish Medicines Formulary).

The independent reviews were cross-referenced by the research manager and 24 criteria with complete agreement from all reviewers were included. For example, all reviewers agreed on the inclusion of “Proton Pump Inhibitor (PPI) for peptic ulcer disease at full therapeutic dosage for >8 weeks”. A total of 31 were excluded. The remaining 25 were re-evaluated in a number of round-table discussions, until full consensus was achieved amongst the group on whether to include or exclude the indicator. From this 25, a further 15 criteria were included, resulting in a list of 39 (see Figure 2).

Appropriate alternative treatment options for each of the selected PIP criteria were identified in the same manner. Initial pharmacological and non- pharmacological treatment alternatives were identified from a review of the current evidence-base which was carried out by a physician (DC) and a pharmacist (MB). Sources such as Clinical Evidence, IMF, BNF, and the National Institute for Health and Clinical Excellence (NICE) were consulted in this process. The recommended alternatives were circulated to members of the panel and the rationale and clinical suitability of each was discussed and reviewed in a number of round table discussions. Where consensus could not be reached amongst the team, or where no appropriate alternative was located, a clinical pharmacologist with an interest in prescribing in older people (DW) was consulted. This process was on-going until full consensus was reached amongst the study team on which pharmacological and non-pharmacological alternatives to include. For all of the 39 criteria, a therapeutic alternative was identified, with non-pharmacological alternatives being identified for 12. This process was on-going over the period of February to September 2011.

Items within the same drug class were grouped into categories, 18 in total. For example, all criteria relating to the use of nonsteroidal anti-inflammatory drugs (NSAIDs) were grouped together. Therapeutic treatment algorithms were compiled, outlining the particular instance of PIP, the reason for concern and the alternative therapy option in each case.

### Patient case identification

Patient cases were used to test the intervention materials compiled during the consensus methodology stage and also to test mechanisms for patient identification. A convenience sample of eight GPs working in a variety of different general practices, involved in a local continuing medical education (CME) discussion group agreed to participate.

To test the patient identification mechanism, GPs were requested to identify five patients (to minimise workload) aged 70 and over from their practice who were taking at least seven repeat prescription items (as polypharmacy is a risk factor for PIP) [5], and print off an anonymised prescription summary for each, detailing medications and diagnoses, prior to a scheduled meeting date. The anonymised prescription summaries were given to the research pharmacist (MB) to review and identify instances of PIP as per those agreed upon during consensus-based methodology. From testing this mechanism, we decided to reduce the inclusion criteria from seven to two repeat items as this increased the pool of potential patients.

In total, 23 cases (who had seven repeat medications) were identified as having at least one PIP. At the CME meeting, all GPs were given a copy of all 23 cases and asked to conduct a hypothetical medicines review of each case using the therapeutic treatment algorithm. The participants recorded the actions they would have taken in each case, and why, and commented on the validity/ relevance of the recommended alternatives, using specifically designed evaluation sheets.

During this process, a total of 31 potentially inappropriate prescriptions were addressed, across nine medication groups. The most common indicator identified was the use of PPIs at full therapeutic dosage for >8 weeks (29%). The GPs were all in agreement that the PPI indicator, indictors pertaining to NSAIDs, the use of long-acting benzodiazepines and therapeutic duplication were all clinically significant. There was debate over the clinical significance of other indictors relating to the use of tricyclic antidepressants (TCAs) and bladder antimuscarinics (see Table 3).

**Table 3:** Summary of patient cases

**Drug/drug group**	**Agreement on clinical significance**	**Most common actions taken**	**Other comments**
PPI	Yes	Reduce dose	While it is clinically significant, it is also a cost control concern
		Stop medication	
Corticosteroids	Yes	Add medication	
Long-term, long-acting benzodiazepines	Yes	Switch to alternative	Patient preference is an important factor. Availability of other services such as cognitive behavioural therapy is an important factor
		Reduce dose	
NSAID	Yes	Stop medication	
		Switch to alternative	
Bladder antimuscarinics	No	Leave unaltered	Patient preference is an important factor. Lack of good alternative options available
		Switch to alternative	
Tricyclic anti-depressants	No	Leave unaltered	Patient preference is an important factor
		Switch to alternative	
		Reduce dose	
Therapeutic duplications	Yes	Stop medication	
		Switch to alternative	
Calcium channel blocker	No	Switch to alternative	
Theophylline	Yes	Stop medication	

Abbreviations: *NSAID* Nonsteroidal anti-inflammatory drug, *PPI* Proton pump inhibitor.

### Focus group

Directly after conducting the hypothetical patient reviews, participating GPs also took part in a focus group to evaluate the materials. The topic guide focused on the chosen PIP drugs, the recommended alternatives and the decisions the participants would have taken in the clinical setting. The focus group was facilitated by two of the research team (MB and BC) and was audio-recorded. The recording was transcribed verbatim and analysed using a thematic analysis.

The focus group highlighted that participants were supportive of the study rationale and there was a general agreement that it was *“very important to do this type of research from a safety point of view.”* P1

The participants reported that the therapeutic treatment algorithms were very useful and that structured provision of concise information was *“much more effective than reams of guidelines.”* P2

They provided comments on which areas of the algorithms needed to be formatted differently to be clear and concise. Consistent with the evaluation sheets, there was some debate between the participants over the inclusion of certain criteria and over some of the recommended alternatives. In particular, the participants felt that there needed to be more focus on the provision of non-pharmacological alternative advice in the algorithms: *“I think some of the recommendations didn’t, eh, weren’t, comprehensive enough, didn’t look at non-drug things.”* P1

Patient preference for specific medications was mentioned a number of times as being a concern for the participants, regardless of whether or not they thought a problem was clinically significant and it was noted that GPs found it difficult to do a hypothetical review. They anticipated that it could be difficult to convince patients to change a medication and highlighted that there needed to be a strong clinical basis to support altering the medications:

“…*have to be able to say to them, by being on this you have an increased risk of whatever.”* P2. In light of this, it was suggested that it would be useful to have information to give to the patients: *“… it’s a good way of helping people, it’s a good negotiating thing, here’s the information…” *P6

### Intervention refinement

The information from the evaluation sheets and the focus group was compiled and revisions were made to the format of the materials. Any of the PIP criteria and alternatives that were debated during the modelling process were discussed again by the research team and a decision on whether to retain or remove the criteria was made. A total of 34 PIP criteria were included in the final list (see Additional file 1 for a full list of the included and excluded criteria) and the treatment algorithms were edited. The finalised algorithms for each PIP medication group were compiled into a manual of treatment algorithms. A web-based platform was also developed. Initially, the website was designed as a resource where GPs could access study information such as the complete manual of treatment algorithms and outcome forms to return to the research team. The website underwent a number of subsequent iterations and became central to the intervention and is described below in more detail.

**Figure 3:**
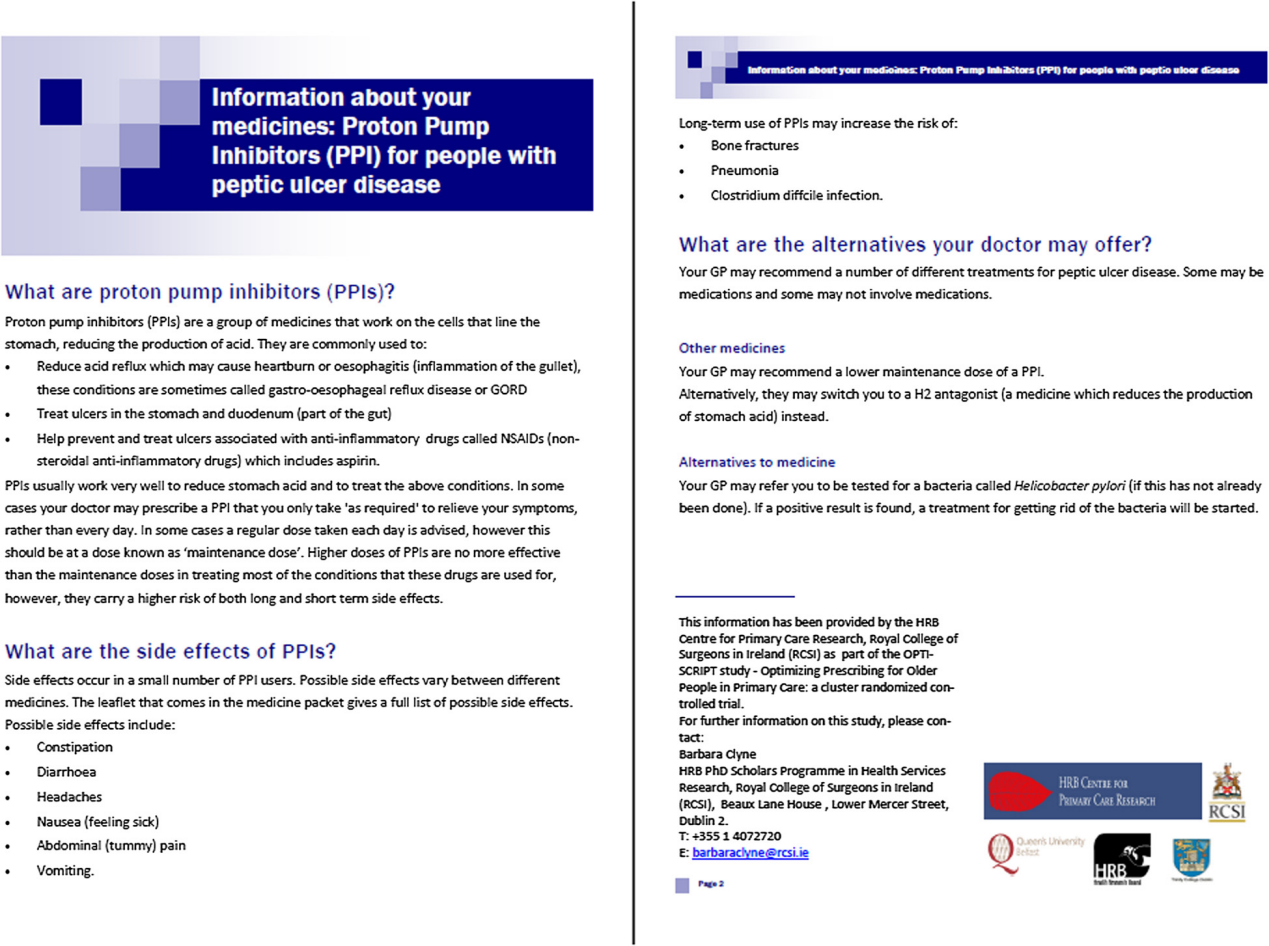
Example of patient information leaflet.

GPs expressed that having information to give to patients would be a useful negotiation tool when it came to discussing medications with patients. The literature review highlighted that patient information leaflets may be helpful in improving patient outcomes and that older patients appreciate being provided with brief, clearly written information leaflets in addition to verbal information from their doctor [42]. As a result, patient information leaflets were developed for the study by the research team (without input from patients). Each leaflet was written in clear and simple language and described what the medication was, why the GP may wish to change the medication and what the alternative treatment alternatives were (see Figure 3 for example). The leaflets were tested with non-clinical researchers to check for clarity and readability.

The findings from the literature were combined with the results from the modelling work to identify the components of the pilot intervention:

1. Academic detailing with a pharmacist

2. Medicines review with web-based treatment algorithms

3. Patient information leaflets tailored to provide specific information for each PIP

### Piloting

The combination of the evidence base and expert opinion in the development stage led to the formatting of the pilot intervention which was tested in the pilot. Five of the CME group GPs agreed to test the intervention material in a real practice setting with a selection of the patients identified previously as having a PIP. The research pharmacist instructed the GPs on the review process and the use of the therapeutic treatment algorithms. The GPs obtained consent from the patients and conducted a real life medicines review using the intervention materials. GPs provided written details of the outcome of the review and participated in a short (5–10 minutes) qualitative semi-structured interview with the study manager (BC). The interviews were audio-recorded, transcribed and a thematic analysis conducted.

A total of 13 patients were invited to take part in the pilot and eight patients consented. Of the five who did not consent, only one refused to take part while the others were unavailable to take part at the time (e.g. had been hospitalised, moved to a nursing home or subsequently died).

During the pilot, eight medicines reviews were conducted, in which a total of ten instances of PIP were addressed, across seven medication groups. Nine out of the ten instances were addressed in the form of either a dose reduction or a discontinuation of a targeted medication. In one case, the prescription was unaltered due to patient preference. More details on the outcomes of the reviews are presented in Table 4. One GP conducted the review successfully over the telephone with the patient. Previously, the research team had not considered this approach but it was an interesting outcome of the pilot.

**Table 4:** Pilot study – outcomes of medicines review

**Patient**	**PIP**	**Outcome of review**
1	PPI	Dose reduction
	TCA and CCB	TCA discontinued
2	PPI	Dose reduction
	Therapeutic duplication - ACE and ARB	ARB discontinued
3	Long term long acting benzodiazepine	Dose reduction
4	PPI	Dose reduction
5	Bladder antimuscarinics and constipation	Left unaltered
6	NSAID and diuretic	NSAID discontinued
7	NSAID and ACE	NSAID discontinued
8	Long term steroid for maintenance therapy in COPD/Asthma	Switched from steroid to other treatment

Abbreviations: *ACEI* Angiotensin-converting-enzyme inhibitor, *ARB* Angiotensin II receptor blockers, *CCB* Calcium channel blocker, *COPD* Chronic obstructive pulmonary disease, *NSAID* Nonsteroidal anti-inflammatory drugs, *PPI* Proton pump inhibitor, *TCA* Tricyclic anti-depressant.

Note: The decision on whether to follow the recommended treatment alternatives will be at the discretion of the GP, weighing up the risks and benefits and patient preference.

### Participant response to the intervention process

The qualitative evaluation of the pilot study indicated that GPs were very positive about both their experience and the patients’ feedback of the review process, and GPs were motivated to alter their prescribing practice:

*“O ya, and she was delighted, I stopped some of her other medications because she was in front of me and I had a bit of time to do it.”* P5.

GPs were also very receptive to the intervention itself:

*“No, I think, I mean, I hope it will be really useful for GPs, I would certainly like it for myself as a GP so that’s the best you can say…”* P4.

GPs in the pilot did not provide feedback on the academic detailing, the finalized content was tested with a GP independent of the focus group to ensure it was clear and informative.

### Participant response to the intervention structure

As in the development stage, it was emphasised that being concise with information was essential:

*“I think in terms of structure, it was better to tailor it more to individual PIPs.”* P6

From the development stage, it emerged that patient information leaflets may be helpful when it came to negotiating patient preference. During the pilot, the patient information leaflets were not entirely successful. While GPs were very pleased to have them, the patients did not necessarily always value them:

*“She wasn’t interested in the information leaflet at all, she was just delighted to stop, so she didn’t take the leaflet home with her at all!”* P5

One GP forgot to use the leaflets, highlighting that leaflets needed to be made more visual to the GPs on screen as a reminder to use them and that the academic detailing process should reinforce their use.

### Barriers identified by participants

Some potential barriers were also identified. GPs felt that the extra time required before seeing the patient to prepare for conducting the review may be an issue, as this involved determining why the prescription had been initiated and by whom:

*“I mean it is time consuming which will be the biggest challenge… it’s nearly a bit of detective work going on, through the notes, trying to work out how did somebody on 16 items get onto some of these drugs.”* P4

Patient preference was another important limiting factor identified by GPs:

*“When I said initially we wanted her to come off it, she said, oh no, I’ve been on that for ages, and I don’t want to come off it.*” P2

### The finalised intervention

Based on the findings from the development process and pilot, the pilot intervention was redesigned and the finalised intervention consisted of academic detailing with a pharmacist, medicines review with web-based therapeutic treatment algorithms and tailored patient information leaflets. Table 5 outlines a comparison of the pilot intervention and the finalised intervention.

**Table 5:** Intervention development

**Intervention component**	**Intervention stage**		
	**Development**	**Pilot**	**Finalised**
Academic detailing with a pharmacist	Small, but potentially important, and relatively consistent effects on prescribing [21].	One brief session delivered in pilot, participants instructed on review process and treatment algorithms	One session (30 minutes) discussing:
			1) PIP
			2) Medicines review
			3) Web-based therapeutic treatment algorithms
Medicines review with web-based therapeutic treatment algorithms	Medicines review identified as a strategy to address PIP [10,28,29]. Pharmacists have role in providing advice and support to enable GPs conduct medicines reviews [30,31].	Structure of treatment algorithms revised	One review per patient conducted using web-based platform which guides GP through process
		Non-pharmacological alternatives added where applicable	Each treatment algorithm has the following structure:
		Barriers of patient preference and time highlighted	1) The individual PIP with reason for concern:
	PIP criteria selected Treatment algorithms to be more structured More focus on non-pharmacological alternatives	Structure of web-based system revised	2) Alternative pharmacological and non-pharmacological treatment options
			3) Background information (where relevant)
Patient information leaflets	Need for information to give to patients highlighted	Patient information leaflets developed, not well utilised in pilot	Patient information leaflets:
			1) Describe the PIP and the reasons as to why it may be inappropriate
	Patient information leaflets may be helpful in improving patient outcomes, older patients appreciate information leaflets in addition to verbal information from their doctor [40].		
			2) Outline the alternative pharmacological and non-pharmacological therapies GPs may offer.

#### *Academic detailing with a pharmacist*

A research pharmacist visits the intervention practices. The aim of the academic detailing session is to educate GPs about the concept of PIP, focusing on the prevalence and consequences of PIP in primary care, and enable them to conduct a medicines review using the intervention materials.

#### *Medicines review with web-based therapeutic treatment algorithms*

GPs in the intervention arm are conducting a medicines review with each participant patient. Each GP is given individual details to log-in during the review. They can select particular patients on repeat medications who were identified as having PIP by the research pharmacist (identified by study ID number only as assigned by the GP) and view the specific treatment algorithm(s) applicable to that patient in pdf format. Each treatment algorithm has the following structure:

Section A: The individual PIP with reason for concern

Section B: Alternative pharmacological and non-pharmacological treatment options

Section C: Background information (where relevant).

Upon completion of the review, the GP is directed to an outcome form page which presents them with a number of tick box options and free text fields to record the outcome of the review. This information is automatically saved to a database for access by the researcher team. Once the review outcome form has been filled in, the medicines review is complete.

#### *Patient information leaflets*

For every alternative therapeutic option, a brief patient information leaflet is available. These leaflets describe the PIP and the reasons as to why it may be inappropriate. They also outline the alternative pharmacological and non-pharmacological therapies the GP may offer instead (see Figure 3).

## Discussion

It is generally accepted that interventions should be developed in a systematic way if they are to be feasible and effective. The purpose of this article was to describe the application of the MRC framework to the development of a primary care intervention targeted at reducing PIP. During the development phase, the pre-existing evidence on the topic of PIP and intervention research was considered by an expert panel and a group of participating GPs to produce a pilot intervention which was then tested. This process allowed the research team to identify areas of the intervention and study design that were in need of further refinement and make the intervention more acceptable to the target study population.

The consensus panel methodology used in the development phase allowed us to select PIP criteria and alternative treatment options that were prevalent and considered clinically relevant to Irish primary care. During the modelling stage it was recommended that the material be more concise and there was more emphasis placed on the addition of non-pharmacological alternatives. The therapeutic treatment algorithms were edited to reflect this.

Patient preference for remaining on certain medications emerged as an important aspect in the development stage. Patient information leaflets were compiled for each of the PIP criteria and recommended alternatives. These leaflets were designed to assist the GP in discussing individual medications with the patient during the medicines review and negotiate changes with them. The leaflets were not universally valued in the pilot but were retained in the finalised intervention as an option for those interested.

Concise information was a key concern of the GPs involved in the modelling process and pilot. With this in mind, the therapeutic treatment algorithms were edited and the web-based platform was designed in such a way that the GPs in the intervention arm would see only the therapeutic treatment algorithm(s) relevant to each patient, making the most efficient use of the consultation time.

The pilot interviews highlighted that there may be ‘detective work’ involved in determining why a particular medication was initiated and by whom. Roughly 30% of the population in Ireland are entitled to free, State-funded GP care and medications. Some 38% of prescriptions for these patients have been found to be initiated by hospital specialists [43]. This highlights concerns around overall responsibility for the prescription and the possibility that the GP may not wish to alter or discontinue a hospital-initiated prescription. To capture this information, an option was incorporated into the outcome form where the GP could tick if the prescription was unaltered for this reason.

The use of qualitative research to help refine the intervention was valuable. The focus group and pilot interviews helped to anticipate and identify barriers to the intervention such as patient preference and the importance of being concise with information. Qualitative interviews will also be conducted at the end of the RCT. We anticipate the qualitative data will provide insight into the intervention delivery and acceptability of the intervention to both GPs and patients.

Although the development process enabled us to make a number of improvements to our intervention, and to achieve a design that is likely to be accepted in the clinical setting, we were constrained by the research context in some areas. Through the modelling and piloting, we were able to test the mechanisms for patient identification and recruitment. The initial mechanism used proved to be quite time consuming and it was proposed that a member of the research team (BC) would become a research agent of the practice to speed up the process. This would also minimize the effort required by the practice staff to recruit patients, which can improve successful recruitment to RCTs [44]. However, the Ethics Committee requested that the patient consent process be done by the practices themselves so the method remained the same as outlined previously.

## Conclusion

The MRC framework provided a systematic way of developing a complex intervention in decreasing PIP in primary care. It provided the opportunity to identify issues and aspects of the intervention that required further development and the pilot phase indicated that the intervention would be well received by GPs, providing support for the implementation of the intervention in clinical practice in Irish primary care. Through the OPTI-SCRIPT study, we expect to determine the effectiveness and acceptability of the intervention in clinical practice.

### Ethical approval

Ethical approval was granted by the Research Ethics Committee of the Irish College of General Practitioners (ICGP).

## Abbreviations

ACEI: Angiotensin-converting-enzyme inhibitor; ARB: Angiotensin II receptor blockers; ACOVE: Assessing care of the vulnerable elder; BC: Barbara clyne; BNF: British national formulary; CCB: Calcium channel blocker; CDSS: Computerised decision support systems); CH: Carmel hughes; CME: Continuing medical education; COPD: Chronic obstructive pulmonary disease; DC: Daniel clear; DW: David Williams; ICGP: Irish College of General Practitioners; IMF: Irish medicines formulary; IPET: Improving prescribing in the elderly tool; MB: Marie Bradley; MRC: Medical Research Council; NICE: National Institute for Health and Clinical Excellence); NSAID: Nonsteroidal anti-inflammatory drug; PIP: (Potentially inappropriate prescribing); PPI: Proton pump inhibitor); RCT: Randomised controlled trial; Rx-PAD: Prescription peer academic detailing); SS: Susan Smith; STOPP: Screening tool of older people’s prescriptions); TCA: Tricyclic anti-depressant; TF: Tom Fahey.

## Competing interests

The authors declare that they have no competing interests.

## Authors’ contributions

All authors conceived the development of the intervention and the study design. BC, SS, TF, CH, MB prepared the protocol and contributed to drafting this paper. DW acted as a clinical expert in pharmacological and therapeutics and reviewed all drafts of this manuscript. RM led the IT design of the study. TF is the principal investigator. Other members of the OPTI-SCRIPT study team are Fiona Boland, Janine Glover and Mary-Claire Kennedy. All authors read and approved the final manuscript.

## Pre-publication history

The pre-publication history for this paper can be accessed here:

http://www.biomedcentral.com/1472-6963/13/307/prepub

## Supplementary Material

Additional file 1 Table of included and excluded criteria. Click here for file

## References

[B1] Gallagher P Barry P O’Mahony D Inappropriate prescribing in the elderly J Clin Pharm Ther 2007 32 113 121 10.1111/j.1365-2710.2007.00793.x 17381661

[B2] Spinewine A Schmader K Barber N Hughes C Lapane K Swine C Hanlon J Appropriate prescribing in elderly people: how well can it be measured and optimised? Lancet 2007 370 173 184 10.1016/S0140-6736(07)61091-5 17630041

[B3] Jano E Aparasu RR Healthcare Outcomes Associated with Beers’ Criteria: A Systematic Review Ann Pharmacother 2007 41 438 447 10.1345/aph.1H473 17311835

[B4] Hamilton H Gallagher P Ryan C Byrne S O’Mahony D Potentially Inappropriate Medications Defined by STOPP Criteria and the Risk of Adverse Drug Events in Older Hospitalized Patients Arch Intern Med 2011 171 1013 1019 10.1001/archinternmed.2011.215 21670370

[B5] Cahir C Fahey T Teeling M Teljeur C Feely J Bennett K Potentially inappropriate prescribing and cost outcomes for older people: a national population study Br J Clin Pharmacol 2010 69 543 552 10.1111/j.1365-2125.2010.03628.x 20573091 PMC2856056

[B6] Gallagher P O’Mahony D STOPP (Screening Tool of Older Persons’ potentially inappropriate Prescriptions) application to acutely ill elderly patients and comparison with Beers’ criteria Age Ageing 2008 37 673 679 10.1093/ageing/afn197 18829684

[B7] Gallagher P Lang P Cherubini A Topinková E Cruz-Jentoft A Montero Errasquín B Mádlová P Gasperini B Baeyens H Baeyens J-P Michel J-P O‘Mahony D Prevalence of potentially inappropriate prescribing in an acutely ill population of older patients admitted to six European hospitals Eur J Clin Pharmacol 2011 67 1175 1188 10.1007/s00228-011-1061-0 21584788

[B8] Byrne S OMahony D Hughes CM Parsons C PS M McCormack B Finn F An evaluation of the inappropriate prescribing in older residents in long term care in the greater Cork and Northern Ireland regions using the STOPP and Beers criteria 2011 Dublin: Centre for ageing research and development in Ireland (CARDI)

[B9] Marcum ZA Handler SM Wright R Hanlon JT Interventions to improve suboptimal prescribing in nursing homes: A narrative review Am J Geriatr Pharmacother 2010 8 183 200 10.1016/j.amjopharm.2010.05.004 20624609 PMC2925103

[B10] Kaur S Mitchell G Vitetta L Roberts MS Interventions that can reduce inappropriate prescribing in the elderly: a systematic review Drugs Aging 2009 26 1013 1028 10.2165/11318890-000000000-00000 19929029

[B11] Loganathan M Singh S Franklin BD Bottle A Majeed A Interventions to optimise prescribing in care homes: systematic review Age Ageing 2011 40 150 162 10.1093/ageing/afq161 21262782

[B12] Majumdar SR Soumerai SB Why most interventions to improve physician prescribing do not seem to work CMAJ 2003 169 30 31 12847036 PMC164939

[B13] Grimshaw JM Shirran L Thomas R Mowatt G Fraser C Bero L Grilli R Harvey E Oxman A O‘Brien MA Changing Provider Behavior: An Overview of Systematic Reviews of Interventions Med Care 2001 39 II2 II45 11583120

[B14] Craig P Dieppe P Macintyre S Michie S Nazareth I Petticrew M Developing and evaluating complex interventions: the new Medical Research Council guidance BMJ 2008 3 37 10.1136/bmj.a1655 PMC2769032 18824488

[B15] Medical Research Council A framework for development and evaluation of RCTs for complex interventions to improve health 2000 London: Medical Research Council

[B16] Forsetlund L Eike M Gjerberg E Vist G Effect of interventions to reduce potentially inappropriate use of drugs in nursing homes: a systematic review of randomised controlled trials BMC Geriatr 2011 11 16 10.1186/1471-2318-11-16 21496345 PMC3108292

[B17] Campbell NC Murray E Darbyshire J Emery J Farmer A Griffiths F Guthrie B Lester H Wilson P Kinmonth AL Designing and evaluating complex interventions to improve health care BMJ 2007 334 455 459 10.1136/bmj.39108.379965.BE 17332585 PMC1808182

[B18] Clyne B Bradley MC Smith SM Hughes CM Motterlini N Clear D McDonnell R Williams D Fahey T Effectiveness of medicines review with web-based pharmaceutical treatment algorithms in reducing potentially inappropriate prescribing in older people in primary care: a cluster randomized trial (OPTI-SCRIPT study protocol) Trials 2013 14 72 10.1186/1745-6215-14-72 23497575 PMC3621288

[B19] Shojania KG Jennings A Mayhew A Ramsey CR Eccles MP Grimshaw J The effects of on-screen, point of care computer reminders on processes and outcomes of care Cochrane Database Syst Rev 2009 3 CD001096 10.1002/14651858.CD001096.pub2 PMC4171964 19588323

[B20] Ostini R Hegney D Jackson C Williamson M Mackson JM Gurman K Hall W Tett SE Systematic Review of Interventions to Improve Prescribing Ann Pharmacother 2009 43 502 513 10.1345/aph.1L488 19261953

[B21] O‘ Brien MA Rogers S Jamtvedt G Oxman AD Odgaard-Jensen J Kristofferson DT Forsetlund L Bainbridge D Freemantle N Davis DA Haynes RB Harvey EL Educational outreach visits: effects on professional practice and health care outcomes Cochrane database syst rev 2007 4 CD000409 17943742 10.1002/14651858.CD000409.pub2 PMC7032679

[B22] Ivers N Jamtvedt G Flottorp S Young J Odgaard-Jensen J French S O‘ Brien MA Johansen M Grimshaw J Oxman AD Audit and feedback: effects on professional practice and healthcare outcomes Cochrane Database Syst Rev 2012 6 CD000259 22696318 10.1002/14651858.CD000259.pub3 PMC11338587

[B23] Castelino RL Bajorek BV Chen TF Targeting suboptimal prescribing in the elderly: a review of the impact of pharmacy services Ann Pharmacother 2009 43 1096 1106 10.1345/aph.1L700 19470856

[B24] Eccles M Grimshaw J Walker A Johnston M Pitts N Changing the behavior of healthcare professionals: the use of theory in promoting the uptake of research findings J Clin Epidemiol 2005 58 107 112 10.1016/j.jclinepi.2004.09.002 15680740

[B25] Bhattacharyya O Reeves S Garfinkel S Zwarenstein M Designing theoretically-informed implementation interventions: Fine in theory, but evidence of effectiveness in practice is needed Implement Sci 2006 1 5 10.1186/1748-5908-1-5 16722583 PMC1436014

[B26] Oxman AD Fretheim A Flottorp S The OFF theory of research utilization J Clin Epidemiol 2005 58 113 116 10.1016/j.jclinepi.2004.10.002 15680741

[B27] Michie S Abraham C Eccles MP Francis J Hardeman W Johnston M Strengthening evaluation and implementation by specifying components of behaviour change interventions: a study protocol Implement Sci 2011 6 10 10.1186/1748-5908-6-10 21299860 PMC3041694

[B28] Green LW Kreuter MW Health Promotion Planning: an educational and ecological approach 3rd edn 1999 Mountain View, CA: Mogfield

[B29] Ramaswamy R Maio V Diamond JJ Talati AR Hartmann CW Arenson C Roehl B Potentially inappropriate prescribing in elderly: assessing doctor knowledge, confidence and barriers J Eval Clin Pract 2011 17 1153 1159 10.1111/j.1365-2753.2010.01494.x 20630004

[B30] Alldred DP Raynor DK Hughes C Barber N Chen TF Spoor P Interventions to optimise prescribing for older people in care homes Cochrane Database Syst Rev 2013 2 CD009095 10.1002/14651858.CD009095.pub2 23450597

[B31] Garcia RM Five ways you can reduce inappropriate prescribing in the elderly: A systematic review J Fam Prac 2006 55 305 312 16608669

[B32] Krska J Gill D Hansford D Pharmacist-supported medication review training for general practitioners: feasibility and acceptability Med Educ 2006 40 1217 1225 10.1111/j.1365-2929.2006.02633.x 17118116

[B33] Drenth-van Maanen AC Van Marum RJ Knol W van der Linden CM Jansen PA Prescribing optimization method for improving prescribing in elderly patients receiving polypharmacy: results of application to case histories by general practitioners Drugs Aging 2009 26 687 701 10.2165/11316400-000000000-00000 19685934

[B34] Craig P Dieppe P Macintyre S Michie S Nazareth I Petticrew M Developing and evaluating complex interventions, new guidance 2008 London: Medical Research Council 10.1136/bmj.a1655 PMC2769032 18824488

[B35] Lewin S Glenton C Oxman AD Use of qualitative methods alongside randomised controlled trials of complex healthcare interventions: methodological study BMJ 2009 339 b3496 10.1136/bmj.b3496 19744976 PMC2741564

[B36] Fick DM Cooper JW Wade WE Waller JL Maclean JR Beers MH Updating the Beers Criteria for Potentially Inappropriate Medication Use in Older Adults: Results of a US Consensus Panel of Experts Arch Intern Med 2003 163 2716 2724 10.1001/archinte.163.22.2716 14662625

[B37] Gallagher P Ryan C Byrne S Kennedy J O‘Mahony D STOPP (Screening Tool of Older Person’s Prescriptions) and START (Screening Tool to Alert doctors to Right Treatment). Consensus validation Int J Clin Pharmacol Ther 2008 46 72 83 18218287 10.5414/cpp46072

[B38] McLeod PJ Huang AR Tamblyn RM Gayton DC Defining inappropriate practices in prescribing for elderly people: a national consensus panel CMAJ 1997 156 385 391 9033421 PMC1226961

[B39] Naugler CT Brymer C Stolee P Arcese ZA Development and validation of an improving prescribing in the elderly tool Can J Clin Pharmacol 2000 7 103 107 10958706

[B40] Wenger NS Roth CP Shekelle P the AI Introduction to the Assessing Care of Vulnerable Elders-3 Quality Indicator Measurement Set JAGS 2007 55 S247 S252 10.1111/j.1532-5415.2007.01328.x 17910544

[B41] Straand J Fetveit A Rognstad S Gjelstad S Brekke M Dalen I A cluster-randomized educational intervention to reduce inappropriate prescription patterns for elderly patients in general practice - The Prescription Peer Academic Detailing (Rx-PAD) study BMC Health Serv Res 2006 6 72 10.1186/1472-6963-6-72 16764734 PMC1525163

[B42] Kenny T Wilson RG Purves IN Clark J Newton LD Newton DP Moseley DV A PIL for every ill? Patient information leaflets (PILs): a review of past, present and future use Fam Pract 1998 15 471 479 10.1093/fampra/15.5.471 9848435

[B43] Feely J Chan R McManus J O’Shea B The influence of hospital-based prescribers on prescribing in general practice Pharmacoeconomics 1999 16 175 181 10.2165/00019053-199916020-00006 10539398

[B44] Page M French S McKenzie J O’Connor D Green S Recruitment difficulties in a primary care cluster randomised trial: investigating factors contributing to general practitioners’ recruitment of patients BMC Med Res Methodol 2011 11 35 10.1186/1471-2288-11-35 21453543 PMC3076278

