# Supplementary material for: Addressing potentially inappropriate prescribing in older patients: development and pilot study of an intervention in primary care (the OPTI-SCRIPT study)
Source: BMC Health Serv Res. 2013 Aug 14;13:307. doi: 10.1186/1472-6963-13-307 (PMC3751793; doi:10.1186/1472-6963-13-307)
Supplement: Additional file 1 — Table of included and excluded criteria. [file 1472-6963-13-307-S1.doc]

| **Criteria** | **Concern** | **Outcome** |
| --- | --- | --- |
| *Cardiovascular* |  |  |
| Digoxin at a long-term dose>125mg/day with impaired renal function | Increased risk of toxicity | Included |
| Loop diuretic for dependent ankle oedema only i.e. no clinical signs of heart failure | No evidence of efficacy, compression hosiery usually more appropriate | Excluded |
| Loop diuretic as first-line monotherapy for hypertension | Safer, more effective alternatives available | Excluded |
| Thiazide diuretic with a history of gout | May exacerbate gout | Included |
| Non-cardioselective betablocker with chronic obstructive pulmonary disease (COPD) | Risk of bronchospasm | Excluded |
| Betablocker in combination with verapamil | Risk of symptomatic heart block | Excluded |
| Betablocker and congestive heart failure | May worsen heart failure | Excluded |
| Use of diltiazem or verapamil with NYHA Class III or IV heart failure | May worsen heart failure | Excluded |
| Calcium channel blockers with chronic constipation | May exacerbate constipation | Included |
| Type I antiarrhythmic agent without an implantable cardioverter defibrillator in heart failure | Increased risk of sudden death | Excluded |
| Use of aspirin and warfarin in combination without histamine H2 receptor antagonist (except cimetidine because of interaction with warfarin) or proton pump inhibitor | high risk of gastro-intestinal bleeding | Included |
| Dipyridamole as monotherapy for cardiovascular secondary prevention | no evidence for efficacy | Excluded |
| Short-acting dipyridamole (Persantin®). Do not consider the long-acting dipyridamole (which has better properties than the short-acting in older adults) except with patients with artificial heart valves | May cause orthostatic hypotension | Excluded |
| Aspirin with a past history of peptic ulcer disease without histamine H2 receptor antagonist or Proton Pump Inhibitor | Risk of bleeding | Excluded |
| Aspirin at dose>150 mg/day | Increased bleeding risk, no evidence for increased efficacy | Included |
| Aspirin with no history of coronary, cerebral or peripheral arterial symptoms or occlusive arterial event | Not indicated | Excluded |
| Aspirin to treat dizziness not clearly attributable to cerebrovascular disease | Not indicated | Excluded |
| Warfarin for first, uncomplicated deep venous thrombosis for longer than 6 months duration | No proven added benefit | Excluded |
| Warfarin for first uncomplicated pulmonary embolus for longer than 12 months duration | No proven benefit | Excluded |
| Aspirin, clopidogrel, dipyridamole or warfarin with concurrent bleeding disorder | Increased risk of bleeding | Excluded |
| The prescription of a long-acting beta-2 agonist inhaler to a patient with asthma who is not also using an inhaled corticosteroid | Need to observe sequential trearment | Excluded |
| Prescription of a potassium salt or potassium sparing diuretic (excluding aldosterone antagonists such as spironolactone) to a patient who is also receiving an ACE inhibitor or AR-II receptor antagonist | Increased risk of hyperkalaemia | Excluded |
| Methyldopa (Aldomet®) | Risk of bradycardia; may exacerbate depression | Excluded |
| Reserpine (Serpalan®, Serpasil®) > 0.25mg | Increased risk of depression, impotence, sedation, and orthostatic hypotension | Excluded |
| Disopyramide (Norpace®, Norpace® CR) | Increased risk of heart failure; Strongly anticholinergic properties | Excluded |
| Ticlopidine (Ticlid®) | No better than aspirin in preventing clotting but considerably more toxic | Excluded |
| Amiodarone (Cordarone®) | Associated with QT interval problems and risk of provoking torsades de pointes | Excluded |
| Cyclandelate (Cyclospasmol®) | Lack of efficacy | Excluded |
| Doxazosin (Cardura®) | Risk of hypotension, dry mouth, and urinary problems. | Excluded |
| Clonidine (Catapres®) | Risk of orthostatic hypotension | Excluded |
| Nifedipine (Procardia®, Adalat®) | Risk of hypotension and constipation | Excluded |
| Ethacrynic acid (Edecrin®) | Risk of hypertension and fluid imbalances | Excluded |
| Ferrous sulfate (iron) | Risk of constipation | Excluded |
| Combination oestrogen and progesterone therapy in patient with Ischaemic heart disease | Increased risk of cardiovascular morbidity and mortality | Excluded |
| *Central nervous system and psychotropic drugs* |  |  |
| Tricyclic antidepressants (TCA’s) with dementia | Risk of worsening cognitive impairment | Included |
| TCA’s with glaucoma | Llikely to exacerbate glaucoma | Included |
| TCA’s with cardiac conductive abnormalities | Pro-arrhythmic effects | Included |
| TCA's and heart block | May worsen heart block | Excluded |
| TCA’s with constipation | May worsen constipation | Included |
| TCA’s with an opiate or calcium channel blocker | Risk of severe constipation | Included |
| TCA’s with prostatism or prior history of urinary retention | Risk of urinary retention | Included |
| TCA's with active metabolites (imipramine, doxepin or amitriptyline) | Strong anticholinergic and sedative properties | Excluded |
| TCA's with history of postural hypotension | May worsen postural hypotension and cause falls | Excluded |
| Nylidrin, niacin or pentoxifylline to treat dementia | No evidence of efficacy | Excluded |
| Meprobamate (Miltown®, Equanil®) | Risk of addiction, sedation | Excluded |
| Methylphenidate for depression | Risk of agitation, stimulation of the CNS and seizures | Excluded |
| Monoamine oxidase inhibitor MAOIs (unless atypical depression is present) |  | Excluded |
| Long-term (i.e. >1 month), long-acting benzodiazepines e.g. chlordiazepoxide, fluazepam, nitrazepam, chlorazepate and benzodiazepines with long-acting metabolites e.g. diazepam | Risk of prolonged sedation, confusion, impaired balance, falls | Included |
| Doses of short-acting benzodiazepines: doses greater than lorazepam (Ativan®), 3 mg; oxazepam (Serax®), 60 mg; alprazolam (Xanax®), 2 mg; temazepam (Restoril®), 15 mg; and triazolam (Halcion®), 0.25 mg | Total daily doses should rarely exceed the suggested maximums | Included |
| Long-term (i.e. >1 month) neuroleptics as long-term hypnotics | Risk of confusion, hypotension, extrapyramidal side effects, falls | Excluded |
| Long-term neuroleptics (>1 month) in those with parkinsonism | May worsen extrapyramidal symptoms | Excluded |
| Phenothiazines in patients with epilepsy | May lower seizure threshold | Excluded |
| Anticholinergics to treat extrapyramidal side-effects of neuroleptic medications | Risk of anticholinergic toxicity | Excluded |
| Anticholinergics | Risk of confusion, urinary retention, consipation, visual disturbance and hypotension | Excluded |
| Selective serotonin re-uptake inhibitors (SSRI’s) with a history of clinically significant hyponatraemia (non-iatrogenic hyponatraemia<130 mmol/l within the previous 2 month) |  | Excluded |
| Prolonged use (>1 week) of first generation antihistamines i.e. diphenydramine, chlorpheniramine, cyclizine, promethazine | Risk of sedation and anticholinergic side effects | Included |
| Ergot mesyloids (Hydergine®) | Have not been shown to be effective in the doses studied | Excluded |
| All barbituates (except phenobarbital) except when used to control seizures | Risk of dependence, falls, confusion | Excluded |
| Amphetamines and anorexic agents | Risk of dependence, hypertension, angina and myocardial infarction | Excluded |
| Daily fluoxetine (Prozac®) | Risk of producing excessive CNS stimulation, sleep disturbances and increasing agitation | Excluded |
| Orphenadrine (Norflex) | Risk of sedation and anticholinergic adverse effects | Excluded |
| Guanethidine (Ismelin®) | Risk of orthostatic hypotension | Excluded |
| Guanadrel (Hylorel®) | Risk of orthostatic hypotension | Excluded |
| Isoxsuprine (Vasodilan®) | Lack of efficacy | Excluded |
| Cimetidine (Tagamet®) | Risk of confusion | Excluded |
| Mesoridazine (Serintil®) | Risk of extrapyramidal side effects | Excluded |
| Thioridazine (Melleril®) | Risk of extrapyramidal side effects | Excluded |
| Amphetamines | Risk of CNS adverse effects | Excluded |
| *Gastro-intestinal* |  |  |
| Diphenoxylate, loperamide or codeine phosphate for treatment of diarrhoea of unknown cause | May exacerbate constipation, may precipitate toxic megacolon in inflammatory bowel disease, may delay recovery in unrecognised gastroenteritis | Excluded |
| Diphenoxylate, loperamide or codeine phosphate for treatment of severe infective gastroenteritis i.e. bloody diarrhoea, high fever or severe systemic toxicity | Risk of exacerbation or protraction of infection | Excluded |
| Prochlorperazine (Stemetil®) or metoclopramide with Parkinsonism | Risk of exacerbating Parkinsonism | Included |
| PPI for peptic ulcer disease at full therapeutic dosage for>8 weeks | Earlier discontinuation or dose reduction for maintenance/prophylactic treatment of peptic ulcer disease, oesophagitis or GORD indicated | Included |
| Anticholinergic antispasmodic drugs with chronic constipation | Risk of exacerbation of constipation | Excluded |
| Gastrointestinal antispasmodic drugs: dicyclomine (Bentyl®), hyoscyamine (Levsin® and Levsinex®), propantheline (Pro-Banthine®), belladonna alkaloids (Donnatal® and others), and clidinium-chlordiazepoxide (Librax®) | Highly anticholinergic, have uncertain effectiveness | Excluded |
| Long-term use of stimulant laxatives: bisacodyl (Dulcolax®), cascara sagrada, and Neoloid® except in the presence of opiate analgesic use | May exacerbate bowel dysfunction | Excluded |
| Trimethobenzamide (Tigan®) | Risk of extrapyramidal side effects | Excluded |
| *Respiratory* |  |  |
| Theophylline as monotherapy for COPD | Risk of adverse effects due to narrow therapeutic index | Included |
| Systemic corticosteroids instead of inhaled corticosteroids for maintenance therapy in moderate-severe COPD | Unnecessary exposure to long-term side-effects systemic steroids | Included |
| Nebulised ipratropium with glaucoma | May exacerbate glaucoma | excluded |
| *Musculoskeletal* |  |  |
| Non-steroidal anti-inflammatory drug (NSAID) with history of peptic ulcer disease or gastro-intestinal bleeding, unless with concurrent histamine H2 receptor antagonist, PPI or misoprostol | Risk of peptic ulcer relapse | Included |
| NSAID with moderate-severe hypertension (moderate: 160/100 mmHg – 179/109 mmHg; severe: 180/110 mmHg) | Risk of exacerbation of hypertension | Excluded |
| NSAID with ACE-inhibitor | Risk of kidney failure, particularly if presence of general arterioscleosis, dehydration or concurrent use of diuretics | Included |
| NSAID with heart failure | Risk of exacerbation of heart failure | Included |
| NSAID with diuretic | May reduce the effect of diuretics and worsen existing heart failure | Included |
| Long-term use of NSAID (>3 months) for relief of mild joint pain in osteoarthtitis | Simple analgesics preferable and usually as effective for pain relief | Included |
| Warfarin and NSAID together | Risk of gastro-intestinal bleeding | Included |
| NSAID with chronic renal failure | Risk of deterioration in renal function | Excluded |
| NSAID with SSRI | Increased risk of GI bleed | Included |
| Long-term NSAID or colchicine for chronic treatment of gout where there is no contraindication to allopurinol | Allopurinol first choice prophylactic drug in gout | Excluded |
| Ketorolac (Toradol®) | Immediate and long-term use should be avoided in older persons, since a significant number have asymptomatic GI pathologic conditions. | Excluded |
| Indomethacin (Indocin®, Indocin® SR) | Risk of CNS adverse effects | Excluded |
| Pentazocine (Talwin®) | Risk of CNS adverse effects, including confusion and hallucinations | Excluded |
| Propoxyphene (Darvon®) and combination products | Poorly tolerated in the elderly | Excluded |
| Muscle relaxants and antispasmodics: methocarbamol (Robaxin®), carisoprodol (Soma), chlorzoxazone (Paraflex®), oxybutynin (Ditropan®). Do not consider the extended-release Ditropan® XL. | Risk of anticholinergic adverse effects, sedation, and weakness | Excluded |
| Meperidine (Demerol®) | Risk of confusion | Excluded |
| Long-term corticosteroids (>3 months) as monotherapy for rheumatoid arthrtitis or osterarthritis | Risk of major systemic corticosteroid side-effects | Included |
| High-dose acetaminophen (paracetamol) (≥3 g/d) and/or with liver disease | Risk of liver toxicity | Excluded |
| Prednisone (or equivalent) > 3 months or longer without bisphosphonate | Increased risk of fracture | Included |
| *Urogenital* |  |  |
| Bladder antimuscarinic drugs with dementia | Risk of increased confusion, agitation | Included |
| Bladder antimuscarinic drugs with chronic glaucoma | Risk of acute exacerbation of glaucoma | Included |
| Bladder antimuscarinic drugs with chronic constipation | Risk of exacerbation of constipation | Included |
| Bladder antimuscarinic drugs with chronic prostatism | Risk of urinary retention | Included |
| Alphablockers in males with frequent incontinence i.e. one or more episodes of incontinence daily | Risk of urinary frequency and worsening of incontinence | excluded |
| Alphablockers with long-term urinary catheter in situ i.e. more than 2 months | Not indicated | Excluded |
| Prescription of a phosphodiesterase type-5 inhibitor, e.g. sildenafil, to a patient who is also receiving a nitrate or nicorandil | Increase hypotensive effect, risk of precipitating angina | Excluded |
| *Endocrine* |  |  |
| Glibenclamide or chlorpropamide with type 2 diabetes mellitus | Risk of prolonged hypoglycaemia | Included |
| Betablockers in those with diabetes mellitus and frequent hypoglycaemic episodes i.e. >1 episode per month | Risk of masking hypoglycaemic symptoms | Excluded |
| Oestrogens with a history of breast cancer or venous thromboembolism | Increased risk of recurrence | Excluded |
| Oestrogens without progestogen in patients with intact uterus | Risk of endometrial cancer | Excluded |
| methyltestosterone (Android®, Testred®, Virilon®) | Risk of prostatic hypertrophy and cardiac problems | Excluded |
| Desiccated thyroid | Concerns about cardiac effects | Excluded |
| *Drugs that adversely affect those prone to falls (1 fall in past 3 months)* |  |  |
| Benzodiazepines | Sedative, may cause reduced sensorium, impair balance | Included |
| Neuroleptic drugs | May cause gait dyspraxia, Parkinsonism | Excluded |
| First generation antihistamines | Sedative, may impair sensorium | Excluded |
| Vasodilator drugs known to cause hypotension in those with persistent postural hypotension i.e. recurrent>20 mmHg drop in systolic blood pressure | Risk of syncope, falls | Excluded |
| Long-term opiates in those with recurrent falls | Risk of drowsiness, postural hypotension, vertigo | Excluded |
| *Analgesic drugs* |  |  |
| Use of long-term powerful opiates e.g. morphine or fentanyl as first line therapy for mild-moderate pain | WHO analgesic ladder not observed | Excluded |
| Regular opiates for more than 2 weeks in those with chronic constipation without concurrent use of laxatives | Risk of severe constipation | Excluded |
| Long-term opiates in those with dementia unless indicted for palliative care or management of moderate/severe chronic pain syndrome | Risk of exacerbation of cognitive impairment | Excluded |
| *Duplicate drug classes* |  |  |
| Any regular duplicate drug class prescription e.g. two concurrent opiates, NSAID’s, SSRI’s, loop diuretics, ACE inhibitors. This excludes duplicate prescribing of drugs that may be required on a PRN basis e.g. Inhaled beta 2 agonists (long and short acting) for asthma or COPD, and opiates for management of breakthrough pain | Optimisation of monotherapy within a single drug class should be observed prior to considering a new class of drug | Included |
| *Other* |  |  |
| Nitrofurantoin (Microdantin®) | Risk of renal impairment | Excluded |
| Mineral Oil | Risk of aspiration | Excluded |
